# Pulmonary Metastasectomy Versus Stereotactic Beam Radiation Therapy for Sarcoma Pulmonary Metastasis: A Retrospective Cohort Study

**DOI:** 10.7759/cureus.96777

**Published:** 2025-11-13

**Authors:** Samuel L Armington, Chris Lamprecht, Adam Lindsay, C. Parker Gibbs

**Affiliations:** 1 Department of Orthopaedic Surgery and Sports Medicine, University of Florida College of Medicine, Gainesville, USA; 2 Department of Orthopedic Surgery, University of Connecticut School of Medicine, Farmington, USA

**Keywords:** bone and soft-tissue sarcoma, orthopedic oncology surgery, pulmonary metastasectomy, sarcoma pulmonary metastasis, stereotactic body radiotherapy (sbrt)

## Abstract

Background

Sarcomas are a diverse group of malignancies that most commonly metastasize to the lungs. Surgical metastasectomy has been considered the gold standard for oligometastatic disease that is technically resectable in medically appropriate patients, while stereotactic body radiotherapy (SBRT) offers a non-surgical alternative that has been shown to be safe and effective. The current literature remains limited in direct comparisons between these treatments.

Methodology

A retrospective cohort study was performed at a single academic center between 2005 and 2024 that included 99 patients with metastatic sarcoma to the lungs. Patients received metastasectomy only (n = 34), SBRT only (n = 28), or both modalities (n = 37). The primary endpoint was overall survival from metastatic diagnosis. Secondary endpoints included treatment-related complications. Multivariable Cox proportional hazards modeling adjusted for treatment modality, age, number of nodules, chemotherapy use, and other prognostic factors.

Results

Before controlling for confounding, metastasectomy was associated with the greatest median survival at 45.5 months, followed by both at 37.5 months and SBRT at 24.5 months. On multivariable analysis, metastasectomy was associated with significantly reduced mortality (hazard ratio (HR) = 0.40, 95% confidence interval (CI) = 0.20-0.82, p = 0.012). SBRT may reduce the risk of mortality, but significance was not reached (HR = 0.86, p = 0.648). Age >70 years (HR = 2.39, p = 0.015) and ≥3 nodules (HR = 2.27, p = 0.008) predicted worse survival. Wide resection of metastases, including microscopic margins, was not associated with statistically significantly reduced mortality when compared to non-wide metastasectomy, with residual microscopic disease (HR = 0.693, p = 0.328). Complication rates were similar between SBRT (17.2%) and metastasectomy (13.4%).

Conclusions

Metastasectomy should remain the preferred, first-line treatment for oligometastatic sarcoma to the lungs in appropriately indicated patients. SBRT remains a safe and effective alternative for patients with unresectable disease or those who are medically inoperable.

## Introduction

Sarcomas are a diverse group of tumors with an incidence of around 7.1 per 100,000 [1]. The lung is the most common site of metastases, and nearly 30% of patients are affected [2]. Patients with metastatic disease have a poor prognosis, with a five-year overall survival estimated at 16% for soft tissue sarcomas and 24% for primary bone sarcomas [3]. Currently, there are three treatment options for patients with pulmonary metastases from sarcoma, namely, resection (metastasectomy), radiation therapy, and chemotherapy.

Surgical metastasectomy has historically been considered the gold standard for treating resectable pulmonary metastatic disease, showing improved survival when all macroscopic disease is resected [4-8]. More recent work has demonstrated that residual microscopic disease reduces survival, and pleural involvement increases the risk of recurrence [9]. Additionally, radiation therapy with stereotactic body radiotherapy (SBRT) has emerged as an effective alternative to surgery relative to survival and local recurrence, particularly among patients with unresectable disease or those deemed unfit to undergo pulmonary resection [10-12].

There is a dearth of studies directly comparing the two treatments. In one series, SBRT was found to have fewer complications than metastasectomy; however, the sample size and propensity matching employed limited the statistical power of the study [13]. Another series included only patients with osteosarcoma and found that the number of lesions and time to metastatic diagnosis were prognostic for survival, but they were unable to identify a difference in survival between surgery and SBRT [14]. A systematic review of retrospective studies found similar mean survival at 46.7 and 47.6 months for metastasectomy and SBRT, respectively [15]. Notably, these studies do not stratify resections by prognostic factors such as residual disease (macroscopic or microscopic) or pleural involvement, which have been shown to affect prognosis [7,9].

In this study, we seek to expand on our previously reported data treating pulmonary metastases with SBRT [11]. With a larger number of patients, we compare metastasectomy to SBRT using multivariable regression to better account for treatment effects.

## Materials and methods

Patients

We conducted a retrospective, cohort study at a single academic medical center. The study was approved by the Institutional Review Board (approval number: 202300394) and was performed in accordance with all requirements. In total, 75 patients were identified via an institutional database with a diagnosis of metastatic sarcoma to the lung who received either SBRT or metastasectomy, in addition to 44 who were previously reported by Lindsay et al. [11]. A total of 20 patients were excluded, with four having incomplete records, six having primary chest wall or pulmonary resections, one having a palliative thoracotomy, and nine having pathology inconsistent with metastatic sarcoma. This left 99 patients for inclusion, of whom 37 received both SBRT and metastasectomy, 34 received only mastectomy, and 28 received only SBRT.

Outcomes and data collection

The primary outcome measure was overall survival from the time of diagnosis of pulmonary metastasis. Secondary outcomes included treatment-related complications. Data points were collected from the electronic medical record via manual chart review and included patient demographics, disease characteristics, treatments received, most recent follow-up, and date of death. The institutional cancer registry and the Social Security Death Index were used to supplement missing mortality data.

SBRT and surgical metastasectomy

Radiation therapy was performed according to institutional protocol, as previously described [11]. Briefly, pulmonary nodules were treated with 50 Gy in five fractions for peripheral lesions and 50 Gy in 10 fractions for central lesions. Pulmonary metastasectomy was performed with wedge resection or lobectomy via an open, thoracoscopic, or robotic-assisted approach chosen at the discretion of the treating surgeon. Resections were classified as wide if all margins were clear of tumor and there was no pleural involvement or lympho-vascular invasion.

Statistical analysis

Baseline characteristics between treatment groups were compared using median (Q1, Q3) for continuous variables and counts (percentages) for categorical variables. Normality was assessed using the Shapiro-Wilk test. Comparison between groups of non-normal continuous variables was performed using the Kruskal-Wallis test (non-parametric). Dunn’s test with Benjamini-Hochberg correction was used for pairwise comparison. Categorical variables were compared with Fisher’s exact test or chi-squared analysis. The Kaplan-Meier method was used to assess the survival of all included patients. Multivariable Cox proportional hazards regression was used to assess the relation between treatments and mortality while controlling for confounding variables, with results expressed as hazard ratios (HRs) and 95% confidence intervals (CIs). Covariates were selected based on clinical relevance and prior reports in the literature. Tied event times were handled with the Breslow method. The proportional hazards assumption was confirmed graphically via plots of scaled Schoenfeld residuals and numerically via Grambsch and Therneau’s method. Violations were categorized into clinically relevant groups. Pairwise comparison between treatment groups was performed using the estimated marginal means method. A p-value of 0.05 was taken as significant. All statistical analysis was performed using the R Survival package, R version 4.4.2 (R Foundation for Statistical Computing, Vienna, Austria).

## Results

Baseline patient and disease characteristics between the three groups are shown in Table 1. Age differed significantly between groups, with SBRT at 68.5 years being significantly greater than metastasectomy at 52.5 years (p < 0.001), and both at 49.0 years (p < 0.001). The proportion of patients receiving chemotherapy was the lowest in the SBRT group at 39.3% compared to 64.9% in the both group, and 64.7% in the metastasectomy group, though this did not reach significance (p = 0.069). There was no significant difference between primary tumor grade, location, or number of nodules treated. The median survival was greatest in the metastasectomy group at 45.5 months, followed by both at 37.5 months, and SBRT at 24.5 months. There was a significant difference in survival with metastasectomy > SBRT (p = 0.013) and both > SBRT (p = 0.006).

**Table 1:** Baseline patient characteristics by treatment group. ^1^: Median (Q1, Q3) or n (%); ^2^: Kruskal-Wallis rank sum test, Fisher’s exact test, or chi-square test. Pairwise comparisons (Dunn’s test): ^*^SBRT > both: age (p < 0.001); ^†^SBRT > metastasectomy: age (p < 0.001); ^‡^: Both > SBRT: survival from metastatic diagnosis (p = 0.006); ^#^: Metastasectomy > SBRT: survival from metastatic diagnosis (p = 0.013).

Variable	Both, n = 37^1^	Metastasectomy, n = 34^1^	SBRT, n = 28^1^	P-value^2^
Age (years)	50.0 (25.0, 67.0)	52.5 (32.0, 65.0)	68.5 (51.5, 78.5)	<0.001
Primary tumor size (cm)	11.0 (7.5, 15.2)	12.0 (9.7, 14.2)	7.0 (6.0, 11.0)	0.062
Location of primary tumor				0.719
Extremity	28 (75.7%)	27 (79.4%)	22 (78.6%)	
Other	7 (18.9%)	3 (8.8%)	4 (14.3%)	
Pelvis	2 (5.4%)	4 (11.8%)	2 (7.1%)	
Pulmonary nodules treated	2.0 (1.0, 2.0)	1.0 (1.0, 3.0)	2.0 (1.0, 3.0)	0.701
Months to metastasis	13.3 (5.0, 19.0)	9.9 (5.5, 21.1)	8.5 (0.0, 32.5)	0.851
Survival from metastatic diagnosis (months)	37.5 (27.2, 79.5)	45.5 (22.0, 69.5)	24.5 (15.4, 36.0)	0.004
Chemotherapy for metastasis	24 (64.9%)	22 (64.7%)	11 (39.3%)	0.069
Tumor grade				0.518
I	4 (10.8%)	0 (0.0%)	2 (7.1%)	
II	5 (13.5%)	4 (11.8%)	4 (14.3%)	
III	27 (73.0%)	29 (85.3%)	22 (78.6%)	
NA	1 (2.7%)	1 (2.9%)	0 (0.0%)	
Histologic diagnosis				-
Chondrosarcoma	0 (0.0%)	2 (5.9%)	1 (3.6%)	
Clear cell chondrosarcoma	0 (0.0%)	0 (0.0%)	1 (3.6%)	
Dedifferentiated chondrosarcoma	2 (5.4%)	1 (2.9%)	0 (0.0%)	
Dedifferentiated liposarcoma	0 (0.0%)	1 (2.9%)	1 (3.6%)	
Dedifferentiated parosteal Osteosarcoma	0 (0.0%)	1 (2.9%)	0 (0.0%)	
Dermatofibrosarcoma	0 (0.0%)	1 (2.9%)	0 (0.0%)	
Ewings sarcoma	4 (10.8%)	0 (0.0%)	0 (0.0%)	
Hemangiopericytoma	1 (2.7%)	0 (0.0%)	1 (3.6%)	
Leiomyosarcoma	4 (10.8%)	2 (5.9%)	5 (17.9%)	
Malignant peripheral nerve Sheath tumor	2 (5.4%)	2 (5.9%)	0 (0.0%)	
Mesenchymal chondrosarcoma (extraskeletal)	0 (0.0%)	1 (2.9%)	0 (0.0%)	
Myxofibrosarcoma	0 (0.0%)	2 (5.9%)	2 (7.1%)	
Myxoid chondrosarcoma (extraskeletal)	0 (0.0%)	1 (2.9%)	0 (0.0%)	
Myxoid liposarcoma	1 (2.7%)	1 (2.9%)	0 (0.0%)	
Osteosarcoma	5 (13.5%)	6 (17.6%)	0 (0.0%)	
Osteosarcoma with chondroblastic features	0 (0.0%)	1 (2.9%)	0 (0.0%)	
Perivascular epithelioid cell tumor	1 (2.7%)	0 (0.0%)	0 (0.0%)	
Pleomorphic sarcoma	1 (2.7%)	1 (2.9%)	0 (0.0%)	
Round cell sarcoma (CIC-DUX4)	1 (2.7%)	0 (0.0%)	0 (0.0%)	
Spindle and pleomorphic cell sarcoma	0 (0.0%)	2 (5.9%)	0 (0.0%)	
Spindle cell sarcoma	1 (2.7%)	2 (5.9%)	1 (3.6%)	
Synovial sarcoma	6 (16.2%)	3 (8.8%)	2 (7.1%)	
Undifferentiated epitheleoid sarcoma	0 (0.0%)	1 (2.9%)	0 (0.0%)	
Undifferentiated pleomorphic sarcoma	8 (21.6%)	3 (8.8%)	14 (50.0%)	

**Figure 1:**
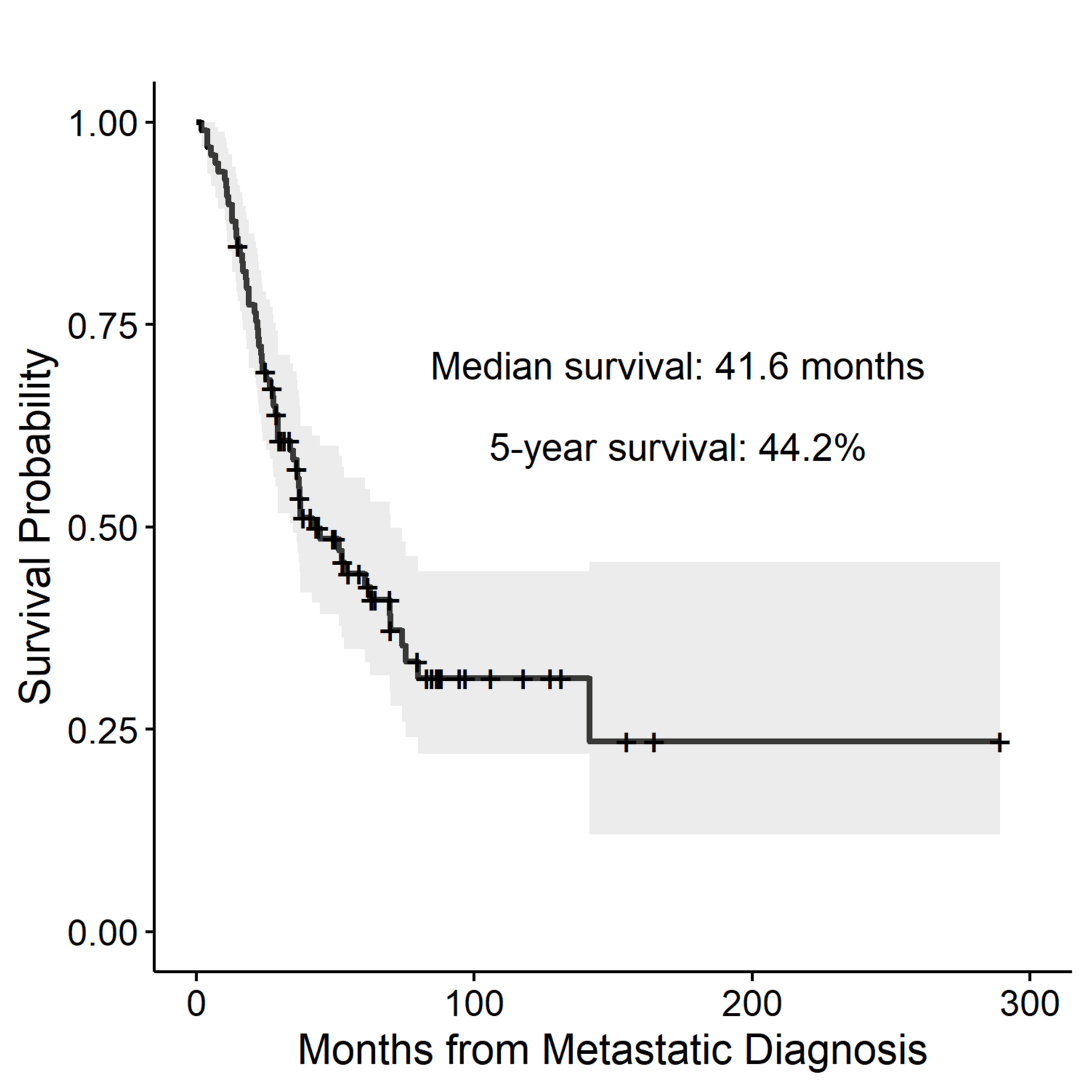
Survival from metastatic diagnosis. The Kaplan-Meier survival analysis showing overall survival from the time of pulmonary metastatic diagnosis for all patients.

The overall survival for all patients from the time of pulmonary metastatic diagnosis is shown in the Kaplan-Meier curve (Figure 1). The median overall survival was 41.6 months, and the five-year survival rate was estimated at 44.2%.

The multivariable Cox proportional hazards model is presented in Table 2 with moderate predictive ability (C-index = 0.685) and significant log-rank (p < 0.001). Developing metastatic disease after 70 years of age was a significant predictor of mortality (HR = 2.39, 95% CI = 1.19-4.81, p = 0.015). Having three or more nodules treated doubled the risk of mortality (HR = 2.27, 95% CI = 1.24-4.14, p = 0.008). The presence of metastatic disease at initial diagnosis was not associated with survival from metastatic diagnosis. Among treatments, chemotherapy was associated with three times the mortality (HR = 3.22, 95% CI = 1.62-6.42, p < 0.001). Metastasectomy was associated with reduced risk of mortality (HR = 0.40, 95% CI = 0.20-0.82, p = 0.012). There was no significant association between SBRT and mortality (HR = 0.86, 95% CI = 0.45-1.64, p = 0.648). On pairwise comparison between patients receiving only metastasectomy versus only SBRT, significance was not reached.

**Table 2:** Multivariable Cox proportional hazards model for predictors of survival from metastatic diagnosis. C-index = 0.685; log-rank p-value <0.001 Pairwise comparison: Metastasectomy only versus SBRT only (HR = 0.491, 95% CI = 0.193–1.252, p = 0.206). CI = confidence interval; HR = hazard ratio; SBRT = stereotactic body radiotherapy

Characteristic	n	HR	95% CI	P-value
Age >70 (years)	25	2.39	1.19–4.81	0.015
3+ nodules treated	24	2.27	1.24–4.14	0.008
Metastasis present at diagnosis	20	1.09	0.60–1.96	0.785
Chemotherapy	57	3.22	1.62–6.42	<0.001
Metastasectomy	71	0.42	0.21–0.85	0.015
SBRT	65	0.86	0.45–1.64	0.648

A multivariable Cox proportional hazards model subclassifying the resection type is shown in Table 3. Again, there was an increased risk for mortality with age >70 years (HR = 2.39, 95% CI = 1.19-4.83, p = 0.015) and having three or more nodules treated (HR = 2.24, 95% CI = 1.20-4.17, p = 0.011). Treatment with chemotherapy increased the hazard for mortality (HR = 3.38, 95% CI = 1.68-6.80, p < 0.001). SBRT was not significantly associated with survival (HR = 0.78, 95% CI = 0.40-1.55, p = 0.485). Notably, a wide resection of the pulmonary metastases was achieved in 55 patients and significantly reduced mortality (HR = 0.33, 95% CI = 0.15-0.74, p = 0.007). There were 17 patients who underwent metastasectomy but did not receive a wide resection. This trended toward reduced mortality, but did not reach significance (HR = 0.48, 95% CI = 0.21-1.09, p = 0.081). Pairwise comparison between wide and other resection margins did not reach significance (HR = 0.693, 95% CI = 0.33-1.45, p = 0.328).

**Table 3:** Multivariable Cox proportional hazards model for predictors of survival from metastatic diagnosis stratified by resection margins. C-index = 0.683; log-rank p-value <0.001. Pairwise comparison: Wide resection – Other resection (HR = 0.693, 95% CI = 0.33–1.45, p = 0.328). CI = confidence interval; HR = hazard ratio; SBRT = stereotactic body radiotherapy

Characteristic	n	HR	95% CI	P-value
Age >70 years	25	2.39	1.19–4.83	0.015
3+ nodules treated	24	2.24	1.20–4.17	0.011
Metastasis present at diagnosis	20	1.02	0.56–1.86	0.949
Chemotherapy	57	3.38	1.68–6.80	<0.001
Metastasectomy wide resection	55	0.33	0.15–0.74	0.007
Metastasectomy other resection	17	0.48	0.21–1.09	0.081
SBRT	65	0.78	0.40–1.55	0.485

Complications for SBRT and metastasectomy are shown in Table 4. The total complication rate was similar between groups. There were 134 metastasectomy procedures and 87 courses of SBRT treatment. The total complication rate was 13.4% with metastasectomy, which included two reoperations. There were 11 pneumothoraxes, three hemothoraxes, and two wound complications. For SBRT, there was a 17.2% overall complication rate, with three patients experiencing cough, two experiencing pain, one rib fracture, eight experiencing pneumonitis, and one experiencing dyspnea.

**Table 4:** Complications noted in the study population. SBRT = stereotactic body radiotherapy; SSI = surgical site infection

Metastasectomy (134), n (%)		SBRT (87), n (%)	
Pneumothorax	11 (8.2%)	Cough	3 (3.4%)
Hemothorax	3 (2.2%)	Pain	2 (2.3%)
SSI/Dehiscence	2 (1.5%)	Dermatitis	0 (0.0%)
Reoperation	2 (1.5%)	Rib fracture	1 (1.1%)
		Pneumonitis	8 (9.2%)
		Dyspnea	1 (1.1%)
Total complications	18 (13.4%)		15 (17.2%)

## Discussion

Our findings support metastasectomy as the standard of care for oligometastatic sarcoma to the lungs when it is technically feasible to resect all macroscopic disease, and the patient is medically appropriate for the surgery. Metastasectomy was found to significantly decrease mortality (HR = 0.42), while SBRT was not associated with a significant decrease in mortality. This may be due in part to unmeasured confounding and sample size limitations; thus, we should not discount SBRT when indicated. Unsurprisingly, advanced age (>70 years) and an increased burden of metastatic disease (3+ nodules) were both associated with decreased survival. Notably, when metastasectomy was subclassified, a significant survival benefit was associated with wide metastasectomy, meaning negative margins and no pleural involvement or lympho-vascular invasion (HR = 0.33). While this statistical benefit did not extend to non-wide resections, the HR was only marginally higher at 0.48 with a p-value of 0.081, which is nearing significance. This is likely related to the smaller number of patients in this group (17 compared to 55 in the wide group). Moreover, the lack of significance between these groups on pairwise comparison suggests the benefit of surgical metastasectomy may be realized, even when a wide resection cannot be achieved. Finally, our median survival of 41.6 months aligns with Tetta et al.’s findings of 46.7 and 47.6 months of survival for metastasectomy and SBRT, respectively [15].

The strengths of this study include the largest sample size of a single study directly comparing metastasectomy to SBRT. To that end, the multivariable regression maintained statistical power while controlling covariates, which has been limited in other reports. This work may also be more generalizable than previous works due to the inclusion of bone and soft tissue sarcomas. The subgroup analysis of microscopic resection margins builds on Welter et al.’s results by incorporating this into a multivariable survival analysis [9]. Finally, the categorization of continuous variables such as age and the number of nodules treated aligns with clinical interpretation.

The primary limitation of this work is the retrospective design and the resulting selection bias, which was mitigated using multivariable survival analysis. Selection bias is manifest in differences in age between treatment groups, with the SBRT group being significantly older than the others. The decreased usage of chemotherapy in the context of decreased survival relative to the other groups suggests increased comorbidity and, thus, unmeasured confounding among patients who underwent SBRT. A prospective comparison between SBRT and metastasectomy would be difficult to perform, given the relatively low incidence and heterogeneity of sarcoma subtypes. Additionally, the lack of clinical equipoise would make randomization ethically prohibitive.

Future work should focus on compiling larger cohorts of patients, which may require collaboration between multiple centers. Larger cohorts would enable a deeper subgroup analysis that was not possible in our study. A Bayesian statistical analysis may help overcome limitations with small sample sizes by incorporating prior studies when appropriate and better quantifying uncertainty within a probabilistic framework, even if external data are not included. Immunotherapies should be another avenue for study, particularly whether their effects may be synergistic with local modalities, and whether they are more effective than traditional chemotherapies. This may help broaden indications for immunotherapies beyond salvage therapy.

## Conclusions

Our results suggest that surgical metastasectomy should remain the treatment of choice for resectable, sarcoma pulmonary metastases in medically appropriate patients. We could not show that pleural involvement or residual microscopic disease significantly negates the benefit of metastasectomy. Likewise, our model did suggest treatment benefit from SBRT, though significance was not reached. Given the unavoidable selection bias and likelihood of unmeasured confounding, along with the body of literature supporting SBRT, it remains an excellent treatment modality for many patients.
